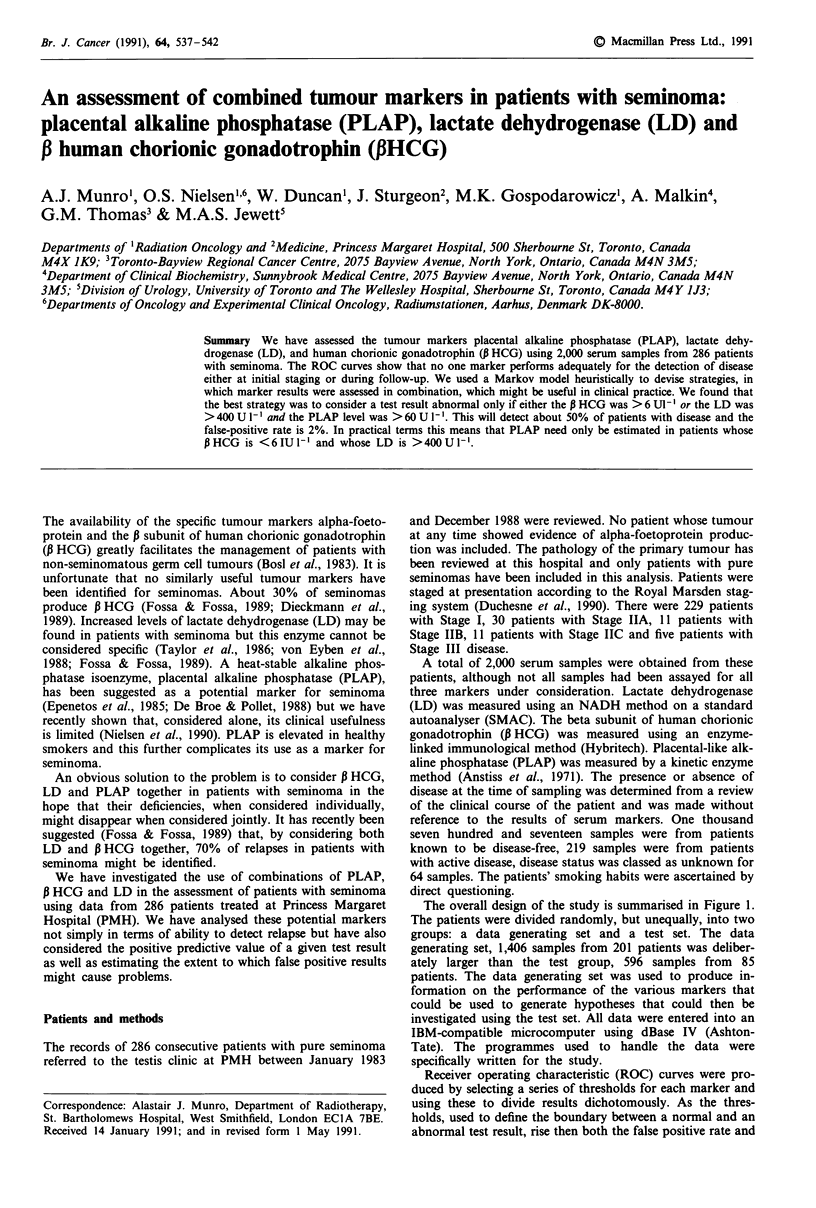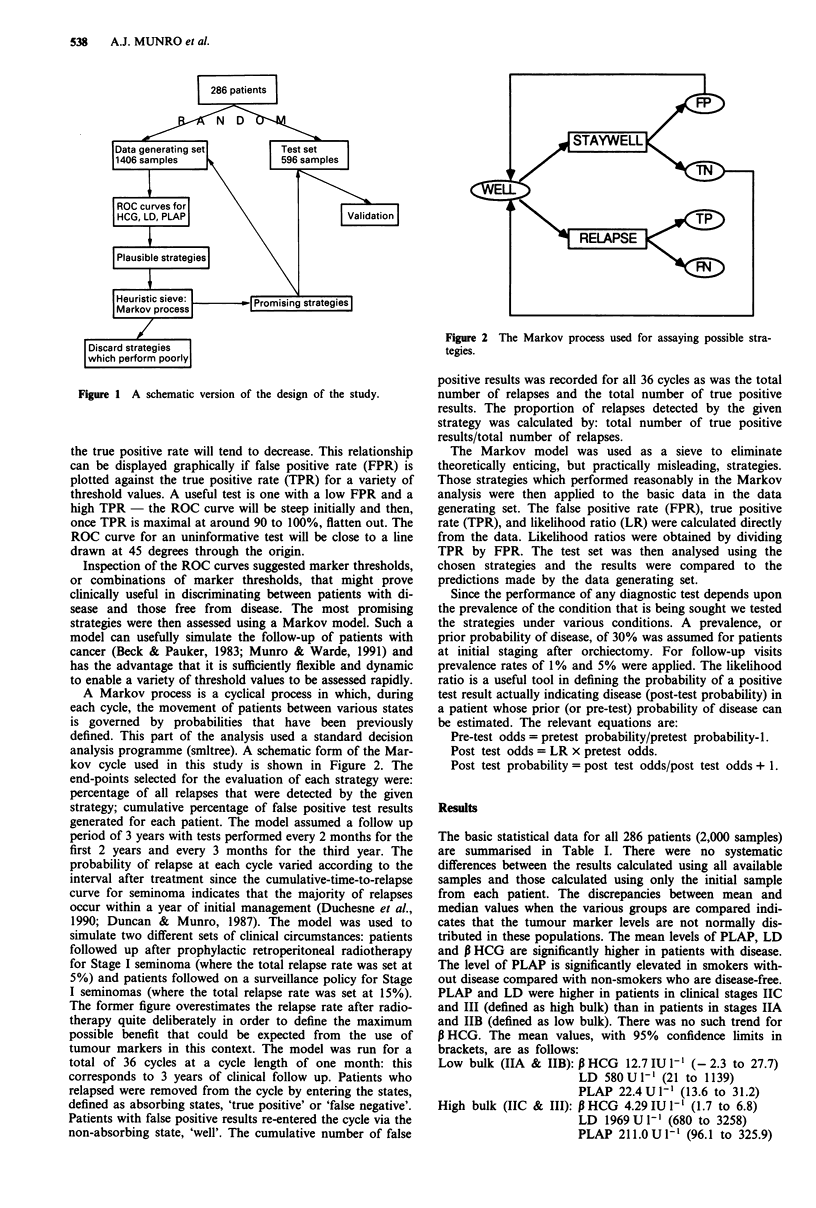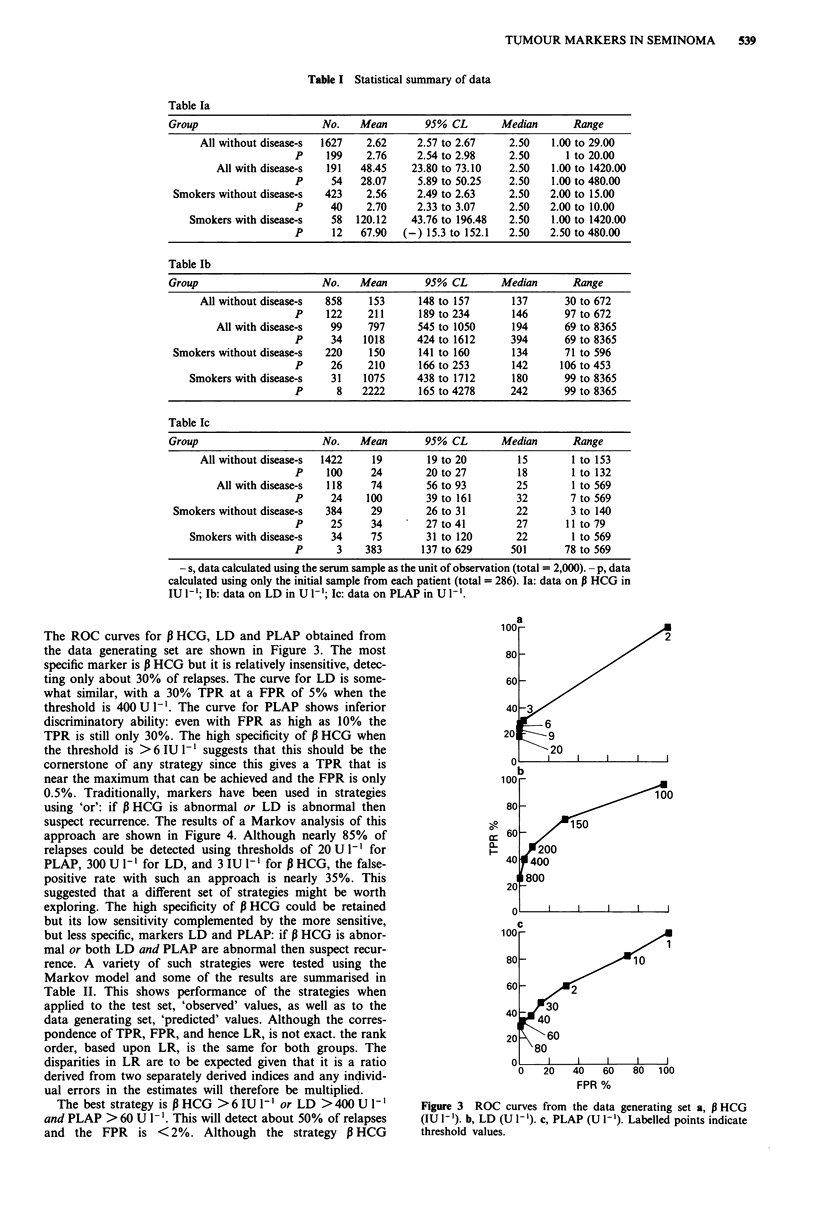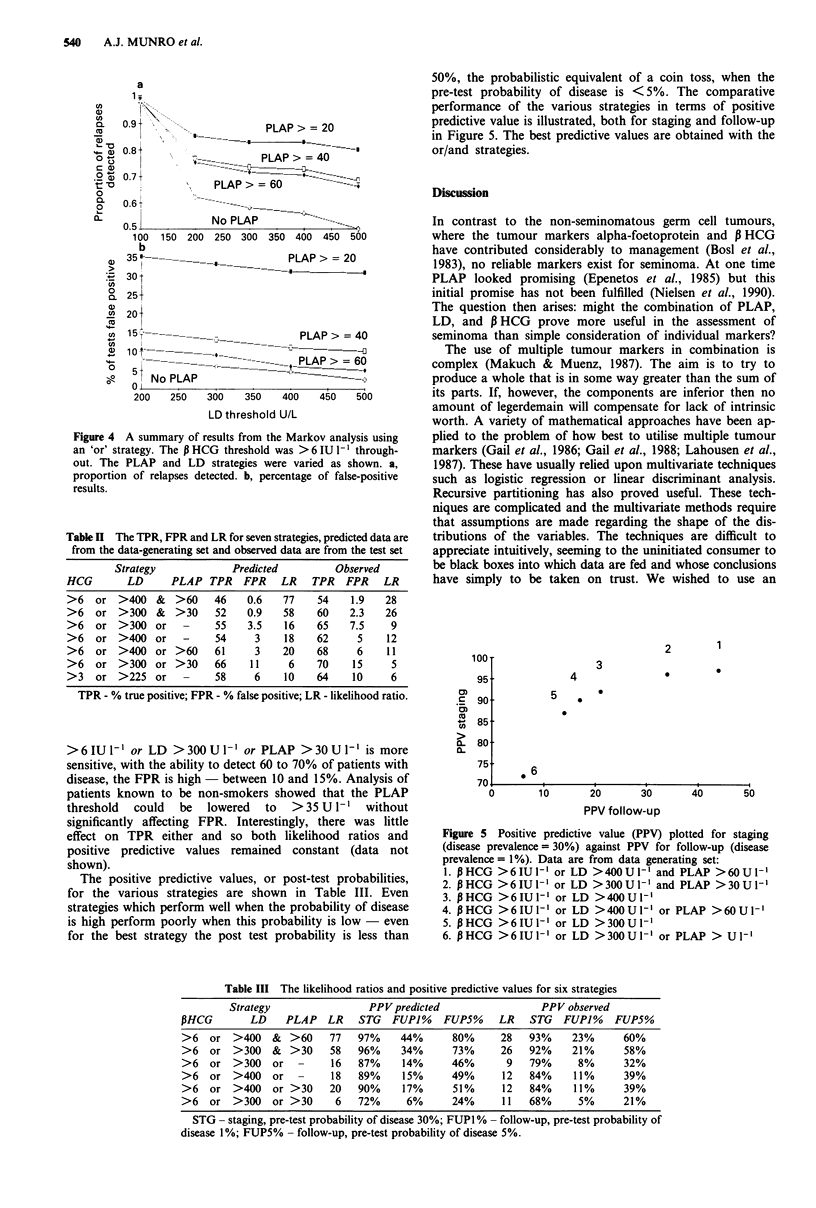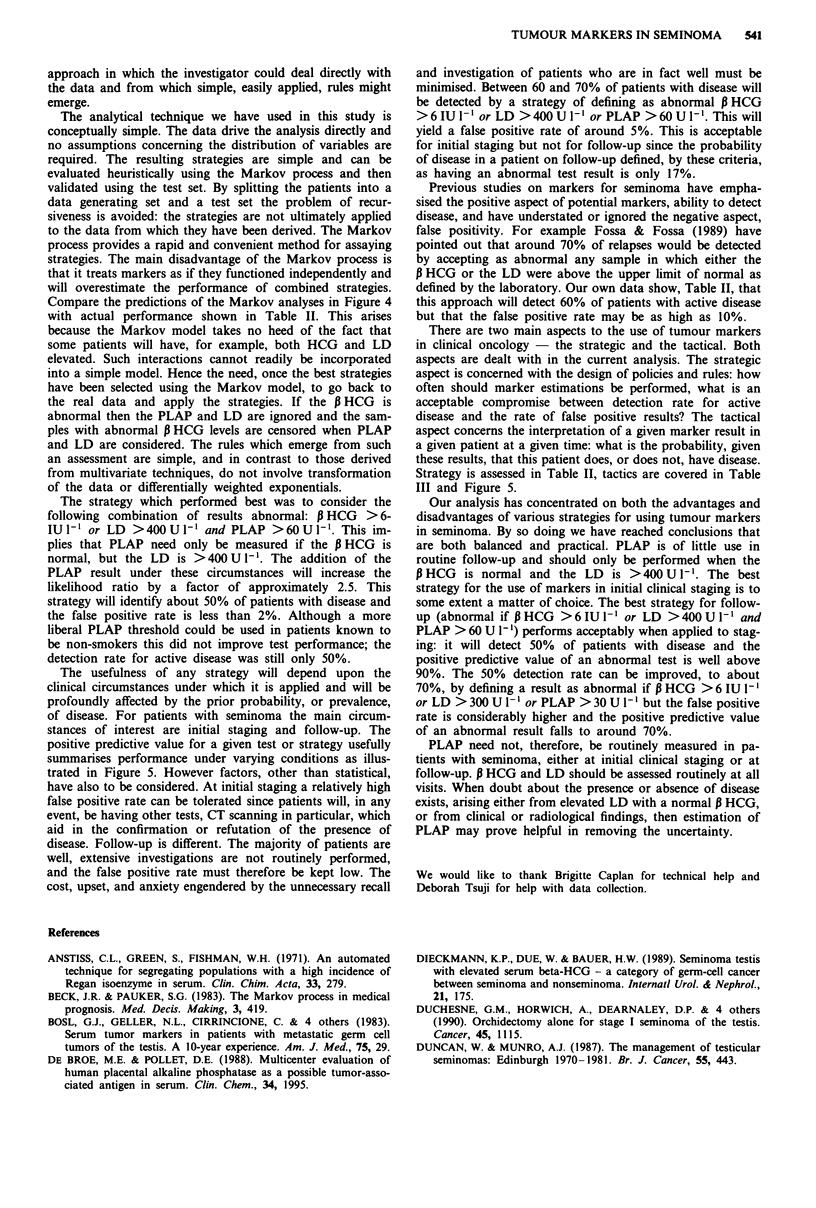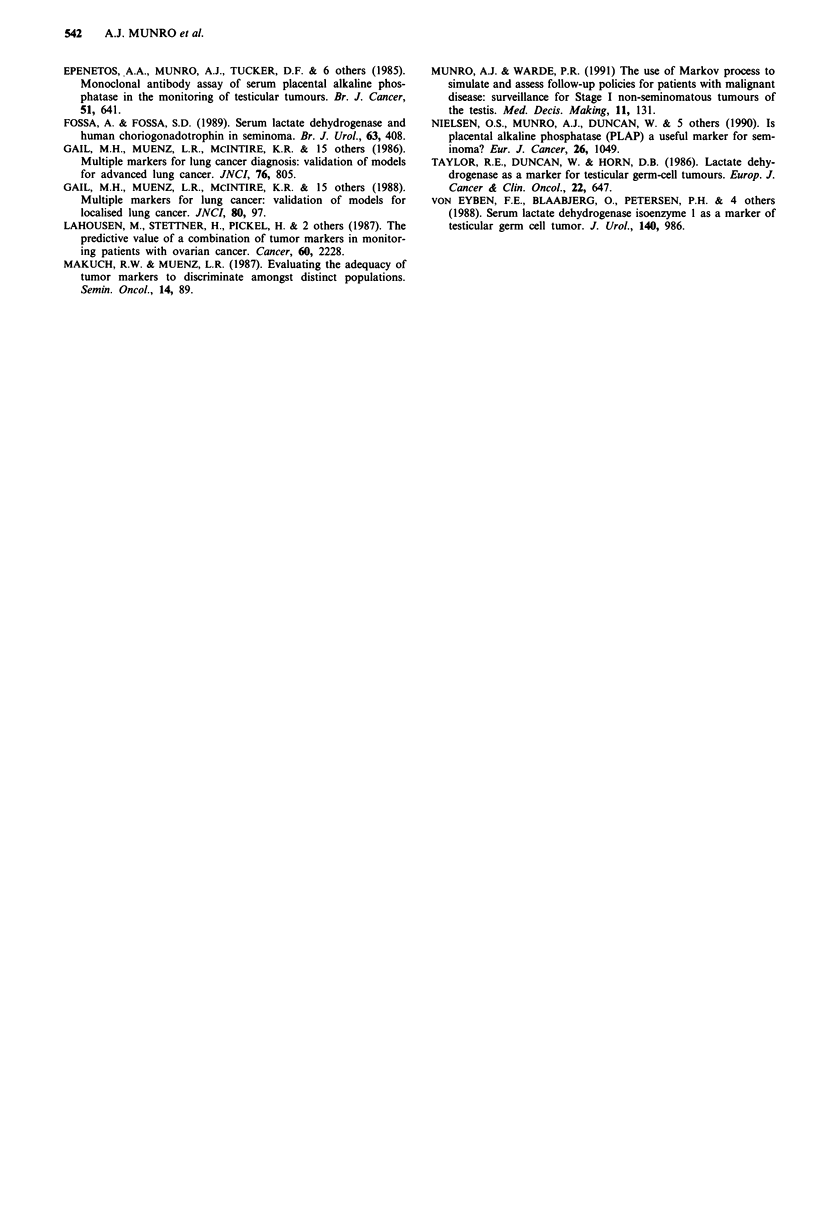# An assessment of combined tumour markers in patients with seminoma: placental alkaline phosphatase (PLAP), lactate dehydrogenase (LD) and beta human chorionic gonadotrophin (beta HCG).

**DOI:** 10.1038/bjc.1991.346

**Published:** 1991-09

**Authors:** A. J. Munro, O. S. Nielsen, W. Duncan, J. Sturgeon, M. K. Gospodarowicz, A. Malkin, G. M. Thomas, M. A. Jewett

**Affiliations:** Department of Radiation Oncology, Princess Margaret Hospital, Toronto, Canada.

## Abstract

We have assessed the tumour markers placental alkaline phosphatase (PLAP), lactate dehydrogenase (LD), and human chorionic gonadotrophin (beta HCG) using 2,000 serum samples from 286 patients with seminoma. The ROC curves show that no one marker performs adequately for the detection of disease either at initial staging or during follow-up. We used a Markov model heuristically to devise strategies, in which marker results were assessed in combination, which might be useful in clinical practice. We found that the best strategy was to consider a test result abnormal only if either the beta HCG was greater than 6 Ul-1 or the LD was greater than 400 U l-1 and the PLAP level was greater than 60 U l-1. This will detect about 50% of patients with disease and the false-positive rate is 2%. In practical terms this means that PLAP need only be estimated in patients whose beta HCG is less than 6 IU l-1 and whose LD is greater than 400 U l-1.


Br. J. Cancer (1991), 64, 537–542 © Macmillan Press Ltd., 1991

### An assessment of combined tumour markers in patients with seminoma: placental alkaline phosphatase (PLAP), lactate dehydrogenase (LD) and β human chorionic gonadotrophin (βHCG)

A.J. Munro[1], O.S. Nielsen[1,6], W. Duncan[1], J. Sturgeon[2], M.K. Gospodarowicz[1], A. Malkin[4], G.M. Thomas[3] & M.A.S. Jewett[5]

*Departments of [1]Radiation Oncology and [2]Medicine, Princess Margaret Hospital, 500 Sherbourne St, Toronto, Canada M4X 1K9; [3]Toronto-Bayview Regional Cancer Centre, 2075 Bayview Avenue, North York, Ontario, Canada M4N 3M5; [4]Department of Clinical Biochemistry, Sunnybrook Medical Centre, 2075 Bayview Avenue, North York, Ontario, Canada M4N 3M5; [5]Division of Urology, University of Toronto and The Wellesley Hospital, Sherbourne St, Toronto, Canada M4Y 1J3; [6]Departments of Oncology and Experimental Clinical Oncology, Radiumstationen, Aarhus, Denmark DK-8000.*

**Summary** We have assessed the tumour markers placental alkaline phosphatase (PLAP), lactate dehydrogenase (LD), and human chorionic gonadotrophin (β HCG) using 2,000 serum samples from 286 patients with seminoma. The ROC curves show that no one marker performs adequately for the detection of disease either at initial staging or during follow-up. We used a Markov model heuristically to devise strategies, in which marker results were assessed in combination, which might be useful in clinical practice. We found that the best strategy was to consider a test result abnormal only if either the β HCG was $>6\ \mathrm{Ul}^{-1}$ *or* the LD was $>400\ \mathrm{U\,l}^{-1}$ *and* the PLAP level was $>60\ \mathrm{U\,l}^{-1}$. This will detect about 50% of patients with disease and the false-positive rate is 2%. In practical terms this means that PLAP need only be estimated in patients whose β HCG is $<6\ \mathrm{IU\,l}^{-1}$ and whose LD is $>400\ \mathrm{U\,l}^{-1}$.

The availability of the specific tumour markers alpha-foetoprotein and the β subunit of human chorionic gonadotrophin (β HCG) greatly facilitates the management of patients with non-seminomatous germ cell tumours (Bosl *et al.*, 1983). It is unfortunate that no similarly useful tumour markers have been identified for seminomas. About 30% of seminomas produce β HCG (Fossa & Fossa, 1989; Dieckmann *et al.*, 1989). Increased levels of lactate dehydrogenase (LD) may be found in patients with seminoma but this enzyme cannot be considered specific (Taylor *et al.*, 1986; von Eyben *et al.*, 1988; Fossa & Fossa, 1989). A heat-stable alkaline phosphatase isoenzyme, placental alkaline phosphatase (PLAP), has been suggested as a potential marker for seminoma (Epenetos *et al.*, 1985; De Broe & Pollet, 1988) but we have recently shown that, considered alone, its clinical usefulness is limited (Nielsen *et al.*, 1990). PLAP is elevated in healthy smokers and this further complicates its use as a marker for seminoma.

An obvious solution to the problem is to consider β HCG, LD and PLAP together in patients with seminoma in the hope that their deficiencies, when considered individually, might disappear when considered jointly. It has recently been suggested (Fossa & Fossa, 1989) that, by considering both LD and β HCG together, 70% of relapses in patients with seminoma might be identified.

We have investigated the use of combinations of PLAP, β HCG and LD in the assessment of patients with seminoma using data from 286 patients treated at Princess Margaret Hospital (PMH). We have analysed these potential markers not simply in terms of ability to detect relapse but have also considered the positive predictive value of a given test result as well as estimating the extent to which false positive results might cause problems.

### Patients and methods

The records of 286 consecutive patients with pure seminoma referred to the testis clinic at PMH between January 1983 and December 1988 were reviewed. No patient whose tumour at any time showed evidence of alpha-foetoprotein production was included. The pathology of the primary tumour has been reviewed at this hospital and only patients with pure seminomas have been included in this analysis. Patients were staged at presentation according to the Royal Marsden staging system (Duchesne *et al.*, 1990). There were 229 patients with Stage I, 30 patients with Stage IIA, 11 patients with Stage IIB, 11 patients with Stage IIC and five patients with Stage III disease.

A total of 2,000 serum samples were obtained from these patients, although not all samples had been assayed for all three markers under consideration. Lactate dehydrogenase (LD) was measured using an NADH method on a standard autoanalyser (SMAC). The beta subunit of human chorionic gonadotrophin (β HCG) was measured using an enzyme-linked immunological method (Hybritech). Placental-like alkaline phosphatase (PLAP) was measured by a kinetic enzyme method (Anstiss *et al.*, 1971). The presence or absence of disease at the time of sampling was determined from a review of the clinical course of the patient and was made without reference to the results of serum markers. One thousand seven hundred and seventeen samples were from patients known to be disease-free, 219 samples were from patients with active disease, disease status was classed as unknown for 64 samples. The patients' smoking habits were ascertained by direct questioning.

The overall design of the study is summarised in Figure 1. The patients were divided randomly, but unequally, into two groups: a data generating set and a test set. The data generating set, 1,406 samples from 201 patients was deliberately larger than the test group, 596 samples from 85 patients. The data generating set was used to produce information on the performance of the various markers that could be used to generate hypotheses that could then be investigated using the test set. All data were entered into an IBM-compatible microcomputer using dBase IV (Ashton-Tate). The programmes used to handle the data were specifically written for the study.

Receiver operating characteristic (ROC) curves were produced by selecting a series of thresholds for each marker and using these to divide results dichotomously. As the thresholds, used to define the boundary between a normal and an abnormal test result, rise then both the false positive rate and

Correspondence: Alastair J. Munro, Department of Radiotherapy, St. Bartholomews Hospital, West Smithfield, London EC1A 7BE.
Received 14 January 1991; and in revised form 1 May 1991.

**Figure 1** A schematic version of the design of the study.

**Figure 2** The Markov process used for assaying possible strategies.

the true positive rate will tend to decrease. This relationship can be displayed graphically if false positive rate (FPR) is plotted against the true positive rate (TPR) for a variety of threshold values. A useful test is one with a low FPR and a high TPR — the ROC curve will be steep initially and then, once TPR is maximal at around 90 to 100%, flatten out. The ROC curve for an uninformative test will be close to a line drawn at 45 degrees through the origin.

Inspection of the ROC curves suggested marker thresholds, or combinations of marker thresholds, that might prove clinically useful in discriminating between patients with disease and those free from disease. The most promising strategies were then assessed using a Markov model. Such a model can usefully simulate the follow-up of patients with cancer (Beck & Pauker, 1983; Munro & Warde, 1991) and has the advantage that it is sufficiently flexible and dynamic to enable a variety of threshold values to be assessed rapidly.

A Markov process is a cyclical process in which, during each cycle, the movement of patients between various states is governed by probabilities that have been previously defined. This part of the analysis used a standard decision analysis programme (smltree). A schematic form of the Markov cycle used in this study is shown in Figure 2. The end-points selected for the evaluation of each strategy were: percentage of all relapses that were detected by the given strategy; cumulative percentage of false positive test results generated for each patient. The model assumed a follow up period of 3 years with tests performed every 2 months for the first 2 years and every 3 months for the third year. The probability of relapse at each cycle varied according to the interval after treatment since the cumulative-time-to-relapse curve for seminoma indicates that the majority of relapses occur within a year of initial management (Duchesne et al., 1990; Duncan & Munro, 1987). The model was used to simulate two different sets of clinical circumstances: patients followed up after prophylactic retroperitoneal radiotherapy for Stage I seminoma (where the total relapse rate was set at 5%) and patients followed on a surveillance policy for Stage I seminomas (where the total relapse rate was set at 15%). The former figure overestimates the relapse rate after radiotherapy quite deliberately in order to define the maximum possible benefit that could be expected from the use of tumour markers in this context. The model was run for a total of 36 cycles at a cycle length of one month: this corresponds to 3 years of clinical follow up. Patients who relapsed were removed from the cycle by entering the states, defined as absorbing states, 'true positive' or 'false negative'. Patients with false positive results re-entered the cycle via the non-absorbing state, 'well'. The cumulative number of false positive results was recorded for all 36 cycles as was the total number of relapses and the total number of true positive results. The proportion of relapses detected by the given strategy was calculated by: total number of true positive results/total number of relapses.

The Markov model was used as a sieve to eliminate theoretically enticing, but practically misleading, strategies. Those strategies which performed reasonably in the Markov analysis were then applied to the basic data in the data generating set. The false positive rate (FPR), true positive rate (TPR), and likelihood ratio (LR) were calculated directly from the data. Likelihood ratios were obtained by dividing TPR by FPR. The test set was then analysed using the chosen strategies and the results were compared to the predictions made by the data generating set.

Since the performance of any diagnostic test depends upon the prevalence of the condition that is being sought we tested the strategies under various conditions. A prevalence, or prior probability of disease, of 30% was assumed for patients at initial staging after orchiectomy. For follow-up visits prevalence rates of 1% and 5% were applied. The likelihood ratio is a useful tool in defining the probability of a positive test result actually indicating disease (post-test probability) in a patient whose prior (or pre-test) probability of disease can be estimated. The relevant equations are:

Pre-test odds = pretest probability/pretest probability-1.

Post test odds = LR × pretest odds.

Post test probability = post test odds/post test odds + 1.

### Results

The basic statistical data for all 286 patients (2,000 samples) are summarised in Table I. There were no systematic differences between the results calculated using all available samples and those calculated using only the initial sample from each patient. The discrepancies between mean and median values when the various groups are compared indicates that the tumour marker levels are not normally distributed in these populations. The mean levels of PLAP, LD and β HCG are significantly higher in patients with disease. The level of PLAP is significantly elevated in smokers without disease compared with non-smokers who are disease-free. PLAP and LD were higher in patients in clinical stages IIC and III (defined as high bulk) than in patients in stages IIA and IIB (defined as low bulk). There was no such trend for β HCG. The mean values, with 95% confidence limits in brackets, are as follows:

Low bulk (IIA & IIB): β HCG 12.7 IU $l^{-1}$ (−2.3 to 27.7)

LD 580 U $l^{-1}$ (21 to 1139)

PLAP 22.4 U $l^{-1}$ (13.6 to 31.2)

High bulk (IIC & III): β HCG 4.29 IU $l^{-1}$ (1.7 to 6.8)

LD 1969 U $l^{-1}$ (680 to 3258)

PLAP 211.0 U $l^{-1}$ (96.1 to 325.9)

**Table I** Statistical summary of data

Table Ia

| Group | No. | Mean | 95% CL | Median | Range |
|---|---|---|---|---|---|
| All without disease-s | 1627 | 2.62 | 2.57 to 2.67 | 2.50 | 1.00 to 29.00 |
| *P* | 199 | 2.76 | 2.54 to 2.98 | 2.50 | 1 to 20.00 |
| All with disease-s | 191 | 48.45 | 23.80 to 73.10 | 2.50 | 1.00 to 1420.00 |
| *P* | 54 | 28.07 | 5.89 to 50.25 | 2.50 | 1.00 to 480.00 |
| Smokers without disease-s | 423 | 2.56 | 2.49 to 2.63 | 2.50 | 2.00 to 15.00 |
| *P* | 40 | 2.70 | 2.33 to 3.07 | 2.50 | 2.00 to 10.00 |
| Smokers with disease-s | 58 | 120.12 | 43.76 to 196.48 | 2.50 | 1.00 to 1420.00 |
| *P* | 12 | 67.90 | (–) 15.3 to 152.1 | 2.50 | 2.50 to 480.00 |

Table Ib

| Group | No. | Mean | 95% CL | Median | Range |
|---|---|---|---|---|---|
| All without disease-s | 858 | 153 | 148 to 157 | 137 | 30 to 672 |
| *P* | 122 | 211 | 189 to 234 | 146 | 97 to 672 |
| All with disease-s | 99 | 797 | 545 to 1050 | 194 | 69 to 8365 |
| *P* | 34 | 1018 | 424 to 1612 | 394 | 69 to 8365 |
| Smokers without disease-s | 220 | 150 | 141 to 160 | 134 | 71 to 596 |
| *P* | 26 | 210 | 166 to 253 | 142 | 106 to 453 |
| Smokers with disease-s | 31 | 1075 | 438 to 1712 | 180 | 99 to 8365 |
| *P* | 8 | 2222 | 165 to 4278 | 242 | 99 to 8365 |

Table Ic

| Group | No. | Mean | 95% CL | Median | Range |
|---|---|---|---|---|---|
| All without disease-s | 1422 | 19 | 19 to 20 | 15 | 1 to 153 |
| *P* | 100 | 24 | 20 to 27 | 18 | 1 to 132 |
| All with disease-s | 118 | 74 | 56 to 93 | 25 | 1 to 569 |
| *P* | 24 | 100 | 39 to 161 | 32 | 7 to 569 |
| Smokers without disease-s | 384 | 29 | 26 to 31 | 22 | 3 to 140 |
| *P* | 25 | 34 | 27 to 41 | 27 | 11 to 79 |
| Smokers with disease-s | 34 | 75 | 31 to 120 | 22 | 1 to 569 |
| *P* | 3 | 383 | 137 to 629 | 501 | 78 to 569 |

– s, data calculated using the serum sample as the unit of observation (total = 2,000). – p, data calculated using only the initial sample from each patient (total = 286). Ia: data on $\beta$ HCG in IU $l^{-1}$; Ib: data on LD in U $l^{-1}$; Ic: data on PLAP in U $l^{-1}$.

The ROC curves for $\beta$ HCG, LD and PLAP obtained from the data generating set are shown in Figure 3. The most specific marker is $\beta$ HCG but it is relatively insensitive, detecting only about 30% of relapses. The curve for LD is somewhat similar, with a 30% TPR at a FPR of 5% when the threshold is 400 U $l^{-1}$. The curve for PLAP shows inferior discriminatory ability: even with FPR as high as 10% the TPR is still only 30%. The high specificity of $\beta$ HCG when the threshold is $>6$ IU $l^{-1}$ suggests that this should be the cornerstone of any strategy since this gives a TPR that is near the maximum that can be achieved and the FPR is only 0.5%. Traditionally, markers have been used in strategies using 'or': if $\beta$ HCG is abnormal *or* LD is abnormal then suspect recurrence. The results of a Markov analysis of this approach are shown in Figure 4. Although nearly 85% of relapses could be detected using thresholds of 20 U $l^{-1}$ for PLAP, 300 U $l^{-1}$ for LD, and 3 IU $l^{-1}$ for $\beta$ HCG, the false-positive rate with such an approach is nearly 35%. This suggested that a different set of strategies might be worth exploring. The high specificity of $\beta$ HCG could be retained but its low sensitivity complemented by the more sensitive, but less specific, markers LD and PLAP: if $\beta$ HCG is abnormal *or* both LD *and* PLAP are abnormal then suspect recurrence. A variety of such strategies were tested using the Markov model and some of the results are summarised in Table II. This shows performance of the strategies when applied to the test set, 'observed' values, as well as to the data generating set, 'predicted' values. Although the correspondence of TPR, FPR, and hence LR, is not exact. the rank order, based upon LR, is the same for both groups. The disparities in LR are to be expected given that it is a ratio derived from two separately derived indices and any individual errors in the estimates will therefore be multiplied.

The best strategy is $\beta$ HCG $>6$ IU $l^{-1}$ *or* LD $>400$ U $l^{-1}$ *and* PLAP $>60$ U $l^{-1}$. This will detect about 50% of relapses and the FPR is $<2\%$. Although the strategy $\beta$ HCG

**Figure 3** ROC curves from the data generating set **a**, $\beta$ HCG (IU $l^{-1}$). **b**, LD (U $l^{-1}$). **c**, PLAP (U $l^{-1}$). Labelled points indicate threshold values.

**Figure 4** A summary of results from the Markov analysis using an 'or' strategy. The βHCG threshold was >6 IU $l^{-1}$ throughout. The PLAP and LD strategies were varied as shown. **a**, proportion of relapses detected. **b**, percentage of false-positive results.

**Table II** The TPR, FPR and LR for seven strategies, predicted data are from the data-generating set and observed data are from the test set

| *Strategy* | | | | | *Predicted* | | | *Observed* | | |
|---|---|---|---|---|---|---|---|---|---|---|
| *HCG* | | *LD* | | *PLAP* | *TPR* | *FPR* | *LR* | *TPR* | *FPR* | *LR* |
| >6 | or | >400 | & | >60 | 46 | 0.6 | 77 | 54 | 1.9 | 28 |
| >6 | or | >300 | & | >30 | 52 | 0.9 | 58 | 60 | 2.3 | 26 |
| >6 | or | >300 | or | – | 55 | 3.5 | 16 | 65 | 7.5 | 9 |
| >6 | or | >400 | or | – | 54 | 3 | 18 | 62 | 5 | 12 |
| >6 | or | >400 | or | >60 | 61 | 3 | 20 | 68 | 6 | 11 |
| >6 | or | >300 | or | >30 | 66 | 11 | 6 | 70 | 15 | 5 |
| >3 | or | >225 | or | – | 58 | 6 | 10 | 64 | 10 | 6 |

TPR - % true positive; FPR - % false positive; LR - likelihood ratio.

>6 IU $l^{-1}$ *or* LD >300 U $l^{-1}$ *or* PLAP >30 U $l^{-1}$ is more sensitive, with the ability to detect 60 to 70% of patients with disease, the FPR is high — between 10 and 15%. Analysis of patients known to be non-smokers showed that the PLAP threshold could be lowered to >35 U $l^{-1}$ without significantly affecting FPR. Interestingly, there was little effect on TPR either and so both likelihood ratios and positive predictive values remained constant (data not shown).

The positive predictive values, or post-test probabilities, for the various strategies are shown in Table III. Even strategies which perform well when the probability of disease is high perform poorly when this probability is low — even for the best strategy the post test probability is less than 50%, the probabilistic equivalent of a coin toss, when the pre-test probability of disease is <5%. The comparative performance of the various strategies in terms of positive predictive value is illustrated, both for staging and follow-up in Figure 5. The best predictive values are obtained with the or/and strategies.

### Discussion

In contrast to the non-seminomatous germ cell tumours, where the tumour markers alpha-foetoprotein and βHCG have contributed considerably to management (Bosl *et al.*, 1983), no reliable markers exist for seminoma. At one time PLAP looked promising (Epenetos *et al.*, 1985) but this initial promise has not been fulfilled (Nielsen *et al.*, 1990). The question then arises: might the combination of PLAP, LD, and βHCG prove more useful in the assessment of seminoma than simple consideration of individual markers?

The use of multiple tumour markers in combination is complex (Makuch & Muenz, 1987). The aim is to try to produce a whole that is in some way greater than the sum of its parts. If, however, the components are inferior then no amount of legerdemain will compensate for lack of intrinsic worth. A variety of mathematical approaches have been applied to the problem of how best to utilise multiple tumour markers (Gail *et al.*, 1986; Gail *et al.*, 1988; Lahousen *et al.*, 1987). These have usually relied upon multivariate techniques such as logistic regression or linear discriminant analysis. Recursive partitioning has also proved useful. These techniques are complicated and the multivariate methods require that assumptions are made regarding the shape of the distributions of the variables. The techniques are difficult to appreciate intuitively, seeming to the uninitiated consumer to be black boxes into which data are fed and whose conclusions have simply to be taken on trust. We wished to use an

**Figure 5** Positive predictive value (PPV) plotted for staging (disease prevalence = 30%) against PPV for follow-up (disease prevalence = 1%). Data are from data generating set:

1. βHCG >6 IU $l^{-1}$ or LD >400 U $l^{-1}$ and PLAP >60 U $l^{-1}$

2. βHCG >6 IU $l^{-1}$ or LD >300 U $l^{-1}$ and PLAP >30 U $l^{-1}$

3. βHCG >6 IU $l^{-1}$ or LD >400 U $l^{-1}$

4. βHCG >6 IU $l^{-1}$ or LD >400 U $l^{-1}$ or PLAP >60 U $l^{-1}$

5. βHCG >6 IU $l^{-1}$ or LD >300 U $l^{-1}$

6. βHCG >6 IU $l^{-1}$ or LD >300 U $l^{-1}$ or PLAP > U $l^{-1}$

**Table III** The likelihood ratios and positive predictive values for six strategies

| *Strategy* | | | | | | *PPV predicted* | | | | *PPV observed* | | |
|---|---|---|---|---|---|---|---|---|---|---|---|---|
| *βHCG* | | *LD* | | *PLAP* | *LR* | *STG* | *FUP1%* | *FUP5%* | *LR* | *STG* | *FUP1%* | *FUP5%* |
| >6 | or | >400 | & | >60 | 77 | 97% | 44% | 80% | 28 | 93% | 23% | 60% |
| >6 | or | >300 | & | >30 | 58 | 96% | 34% | 73% | 26 | 92% | 21% | 58% |
| >6 | or | >300 | or | – | 16 | 87% | 14% | 46% | 9 | 79% | 8% | 32% |
| >6 | or | >400 | or | – | 18 | 89% | 15% | 49% | 12 | 84% | 11% | 39% |
| >6 | or | >400 | or | >30 | 20 | 90% | 17% | 51% | 12 | 84% | 11% | 39% |
| >6 | or | >300 | or | >30 | 6 | 72% | 6% | 24% | 11 | 68% | 5% | 21% |

STG – staging, pre-test probability of disease 30%; FUP1% – follow-up, pre-test probability of disease 1%; FUP5% – follow-up, pre-test probability of disease 5%.

approach in which the investigator could deal directly with the data and from which simple, easily applied, rules might emerge.

The analytical technique we have used in this study is conceptually simple. The data drive the analysis directly and no assumptions concerning the distribution of variables are required. The resulting strategies are simple and can be evaluated heuristically using the Markov process and then validated using the test set. By splitting the patients into a data generating set and a test set the problem of recursiveness is avoided: the strategies are not ultimately applied to the data from which they have been derived. The Markov process provides a rapid and convenient method for assaying strategies. The main disadvantage of the Markov process is that it treats markers as if they functioned independently and will overestimate the performance of combined strategies. Compare the predictions of the Markov analyses in Figure 4 with actual performance shown in Table II. This arises because the Markov model takes no heed of the fact that some patients will have, for example, both HCG and LD elevated. Such interactions cannot readily be incorporated into a simple model. Hence the need, once the best strategies have been selected using the Markov model, to go back to the real data and apply the strategies. If the $\beta$HCG is abnormal then the PLAP and LD are ignored and the samples with abnormal $\beta$HCG levels are censored when PLAP and LD are considered. The rules which emerge from such an assessment are simple, and in contrast to those derived from multivariate techniques, do not involve transformation of the data or differentially weighted exponentials.

The strategy which performed best was to consider the following combination of results abnormal: $\beta$HCG $>$6 IU $l^{-1}$ *or* LD $>$400 U $l^{-1}$ *and* PLAP $>$60 U $l^{-1}$. This implies that PLAP need only be measured if the $\beta$HCG is normal, but the LD is $>$400 U $l^{-1}$. The addition of the PLAP result under these circumstances will increase the likelihood ratio by a factor of approximately 2.5. This strategy will identify about 50% of patients with disease and the false positive rate is less than 2%. Although a more liberal PLAP threshold could be used in patients known to be non-smokers this did not improve test performance; the detection rate for active disease was still only 50%.

The usefulness of any strategy will depend upon the clinical circumstances under which it is applied and will be profoundly affected by the prior probability, or prevalence, of disease. For patients with seminoma the main circumstances of interest are initial staging and follow-up. The positive predictive value for a given test or strategy usefully summarises performance under varying conditions as illustrated in Figure 5. However factors, other than statistical, have also to be considered. At initial staging a relatively high false positive rate can be tolerated since patients will, in any event, be having other tests, CT scanning in particular, which aid in the confirmation or refutation of the presence of disease. Follow-up is different. The majority of patients are well, extensive investigations are not routinely performed, and the false positive rate must therefore be kept low. The cost, upset, and anxiety engendered by the unnecessary recall and investigation of patients who are in fact well must be minimised. Between 60 and 70% of patients with disease will be detected by a strategy of defining as abnormal $\beta$HCG $>$6 IU $l^{-1}$ *or* LD $>$400 U $l^{-1}$ *or* PLAP $>$60 U $l^{-1}$. This will yield a false positive rate of around 5%. This is acceptable for initial staging but not for follow-up since the probability of disease in a patient on follow-up defined, by these criteria, as having an abnormal test result is only 17%.

Previous studies on markers for seminoma have emphasised the positive aspect of potential markers, ability to detect disease, and have understated or ignored the negative aspect, false positivity. For example Fossa & Fossa (1989) have pointed out that around 70% of relapses would be detected by accepting as abnormal any sample in which either the $\beta$HCG or the LD were above the upper limit of normal as defined by the laboratory. Our own data show, Table II, that this approach will detect 60% of patients with active disease but that the false positive rate may be as high as 10%.

There are two main aspects to the use of tumour markers in clinical oncology — the strategic and the tactical. Both aspects are dealt with in the current analysis. The strategic aspect is concerned with the design of policies and rules: how often should marker estimations be performed, what is an acceptable compromise between detection rate for active disease and the rate of false positive results? The tactical aspect concerns the interpretation of a given marker result in a given patient at a given time: what is the probability, given these results, that this patient does, or does not, have disease. Strategy is assessed in Table II, tactics are covered in Table III and Figure 5.

Our analysis has concentrated on both the advantages and disadvantages of various strategies for using tumour markers in seminoma. By so doing we have reached conclusions that are both balanced and practical. PLAP is of little use in routine follow-up and should only be performed when the $\beta$HCG is normal and the LD is $>$400 U $l^{-1}$. The best strategy for the use of markers in initial clinical staging is to some extent a matter of choice. The best strategy for follow-up (abnormal if $\beta$HCG $>$6 IU $l^{-1}$ *or* LD $>$400 U $l^{-1}$ *and* PLAP $>$60 U $l^{-1}$) performs acceptably when applied to staging: it will detect 50% of patients with disease and the positive predictive value of an abnormal test is well above 90%. The 50% detection rate can be improved, to about 70%, by defining a result as abnormal if $\beta$HCG $>$6 IU $l^{-1}$ *or* LD $>$300 U $l^{-1}$ *or* PLAP $>$30 U $l^{-1}$ but the false positive rate is considerably higher and the positive predictive value of an abnormal result falls to around 70%.

PLAP need not, therefore, be routinely measured in patients with seminoma, either at initial clinical staging or at follow-up. $\beta$HCG and LD should be assessed routinely at all visits. When doubt about the presence or absence of disease exists, arising either from elevated LD with a normal $\beta$HCG, or from clinical or radiological findings, then estimation of PLAP may prove helpful in removing the uncertainty.

We would like to thank Brigitte Caplan for technical help and Deborah Tsuji for help with data collection.

### References

ANSTISS, C.L., GREEN, S., FISHMAN, W.H. (1971). An automated technique for segregating populations with a high incidence of Regan isoenzyme in serum. *Clin. Chim. Acta*, **33**, 279.

BECK, J.R. & PAUKER, S.G. (1983). The Markov process in medical prognosis. *Med. Decis. Making*, **3**, 419.

BOSL, G.J., GELLER, N.L., CIRRINCIONE, C. & 4 others (1983). Serum tumor markers in patients with metastatic germ cell tumors of the testis. A 10-year experience. *Am. J. Med.*, **75**, 29.

DE BROE, M.E. & POLLET, D.E. (1988). Multicenter evaluation of human placental alkaline phosphatase as a possible tumor-associated antigen in serum. *Clin. Chem.*, **34**, 1995.

DIECKMANN, K.P., DUE, W. & BAUER, H.W. (1989). Seminoma testis with elevated serum beta-HCG – a category of germ-cell cancer between seminoma and nonseminoma. *Internatl Urol. & Nephrol.*, **21**, 175.

DUCHESNE, G.M., HORWICH, A., DEARNALEY, D.P. & 4 others (1990). Orchidectomy alone for stage I seminoma of the testis. *Cancer*, **45**, 1115.

DUNCAN, W. & MUNRO, A.J. (1987). The management of testicular seminomas: Edinburgh 1970–1981. *Br. J. Cancer*, **55**, 443.

EPENETOS, A.A., MUNRO, A.J., TUCKER, D.F. & 6 others (1985). Monoclonal antibody assay of serum placental alkaline phosphatase in the monitoring of testicular tumours. *Br. J. Cancer*, **51**, 641.

FOSSA, A. & FOSSA, S.D. (1989). Serum lactate dehydrogenase and human choriogonadotrophin in seminoma. *Br. J. Urol.*, **63**, 408.

GAIL, M.H., MUENZ, L.R., MCINTIRE, K.R. & 15 others (1986). Multiple markers for lung cancer diagnosis: validation of models for advanced lung cancer. *JNCI*, **76**, 805.

GAIL, M.H., MUENZ, L.R., MCINTIRE, K.R. & 15 others (1988). Multiple markers for lung cancer: validation of models for localised lung cancer. *JNCI*, **80**, 97.

LAHOUSEN, M., STETTNER, H., PICKEL, H. & 2 others (1987). The predictive value of a combination of tumor markers in monitoring patients with ovarian cancer. *Cancer*, **60**, 2228.

MAKUCH, R.W. & MUENZ, L.R. (1987). Evaluating the adequacy of tumor markers to discriminate amongst distinct populations. *Semin. Oncol.*, **14**, 89.

MUNRO, A.J. & WARDE, P.R. (1991) The use of Markov process to simulate and assess follow-up policies for patients with malignant disease: surveillance for Stage I non-seminomatous tumours of the testis. *Med. Decis. Making*, **11**, 131.

NIELSEN, O.S., MUNRO, A.J., DUNCAN, W. & 5 others (1990). Is placental alkaline phosphatase (PLAP) a useful marker for seminoma? *Eur. J. Cancer*, **26**, 1049.

TAYLOR, R.E., DUNCAN, W. & HORN, D.B. (1986). Lactate dehydrogenase as a marker for testicular germ-cell tumours. *Europ. J. Cancer & Clin. Oncol.*, **22**, 647.

VON EYBEN, F.E., BLAABJERG, O., PETERSEN, P.H. & 4 others (1988). Serum lactate dehydrogenase isoenzyme 1 as a marker of testicular germ cell tumor. *J. Urol.*, **140**, 986.